# Prognosis Evaluation in Patients with Hepatocellular Carcinoma after Hepatectomy: Comparison of BCLC, TNM and Hangzhou Criteria Staging Systems

**DOI:** 10.1371/journal.pone.0103228

**Published:** 2014-08-18

**Authors:** Chang Liu, Li-gen Duan, Wu-sheng Lu, Lu-nan Yan, Guang-qin Xiao, Li Jiang, Jian Yang, Jia-yin Yang

**Affiliations:** 1 Department of Liver Surgery, West China Hospital, Sichuan University, Chengdu, China; 2 Institute of Interventional Radiology, West China Hospital, Sichuan University, Chengdu, China; 3 Department of Emergency, West China Hospital, Sichuan University, Chengdu, China; University of Texas MD Anderson Cancer Center, United States of America

## Abstract

**Purpose:**

This study is to evaluate the Hangzhou criteria (HC) for patients with HCC undergoing surgical resection and to identify whether this staging system is superior to other staging systems in predicting the survival of resectable HCC.

**Method:**

774 HCC patients underwent surgical resection between 2007 and 2009 in West China Hospital were enrolled retrospectively. Predictors of survival were identified using the Kaplan–Meier method and the Cox model. The disease state was staged by the HC, as well as by the TNM and BCLC staging systems. Prognostic powers were quantified using a linear trend χ2 test, c-index, and the likelihood ratio (LHR) χ2 test and correlated using Cox's regression model adjusted using the Akaike information criterion (AIC).

**Results:**

Serum AFP level (P = 0.02), tumor size (P<0.001), tumor number (P<0.001), portal vein invasion (P<0.001), hepatic vein invasion (P<0.001), tumor differentiation (P<0.001), and distant organ (P = 0.016) and lymph node metastasis (P<0.001) were identified as independent risk factors of survival after resection by multivariate analysis. The comparison of the different staging system results showed that BCLC had the best homogeneity (likelihood ratio χ^2^ test 151.119, P<0.001), the TNM system had the best monotonicity of gradients (linear trend χ^2^ test 137.523, P<0.001), and discriminatory ability was the highest for the BCLC (the AUCs for 1-year mortality were 0.759) and TNM staging systems (the AUCs for 3-, and 5-year mortality were 0.738 and 0.731, respectively). However, based on the c-index and AIC, the HC was the most informative staging system in predicting survival (c-index 0.6866, AIC 5924.4729).

**Conclusions:**

The HC can provide important prognostic information after surgery. The HC were shown to be a promising survival predictor in a Chinese cohort of patients with resectable HCC.

## Introduction

Hepatocellular carcinoma (HCC) is the fifth most common carcinoma in the world; over half a million cases occur per year, and a gradual increase in its annual incidence has been reported in recent years [1], [2]. Currently, HCC is also the third leading cause of cancer deaths [3] and the main cause of death in cirrhotic patients [4]. HCC's worldwide distribution is heterogeneous and closely related to different risk factors. The incidence is high in China because of a chronic hepatitis B virus (HBV) carrier rate of greater than 10% among the Chinese population [5]. Despite the several treatment options available, such as radiofrequency, transarterial therapy, chemotherapy and radiotherapy, surgical resection is the preferred treatment for HCC; the long-term survival after surgical resection has improved in recent years because of earlier tumor diagnoses and better surgical management.

It is difficult to establish a standard staging system that feasibly predicts survival in all HCC patients [6], [7]; HCC's disease pattern is also heterogeneous in its molecular and clinicopathological features, with diverse etiologies [8], and there are various treatment modalities among different centers. Thus, various staging systems have been developed. Among the several staging systems, the Tumor-Node-Metastasis (TNM) system is one of the most widely accepted, and the 7th edition was published by the American Joint Committee on Cancer (AJCC) in 2009 [9]. The major modification from the 6th to the 7th edition was the separation of the T3 stage into T3a and T3b; this change indicates major vascular invasion of portal or hepatic veins as an important predictive factor for prognosis. The TNM staging system is based on postoperative pathology results; therefore, its application has been limited because most patients with HCC are at an advanced stage that is surgically unresectable at the time of diagnosis. However, Chun et al. reported that the 7th edition AJCC staging system provided better prognostic power than the 6th for patients with HCC based on radiological information, but the prognostic power was worse than that of the BCLC system. The BCLC system was first presented in 1999 [10] and updated in 2010 [11]; it is the only system that provides treatment recommendations for each stage based on the best treatment strategies currently available. The BCLC staging classification has been externally validated in the U.S. [12], Europe [13], and Taiwan [13] and endorsed by both the European Association for the Study of the Liver (EASL) [14] and the American Association for the Study of Liver Diseases (AASLD) [14]. BCLC has demonstrated better survival stratification and prognosis prediction than other staging systems, such as Okuda, CLIP, CUPI, TNM and the JIS classification [15], and has been proposed as the best available prognostic system [6]. However, one study found that the BCLC system was less informative than the GRETCH and CLIP classifications when ranked using Harrell's c-index, the likelihood ratio, and the Akaike information criterion (AIC). The Hangzhou criteria (HC) were first established by Zheng et al. [16] in 2008, HCC patients meeting Hangzhou criteria must fulfill one of the two following items: (a) Total tumor diameter less than or equal to 8 cm; (b) total tumor diameter more than 8 cm, with histopathologic grade I or II and preoperative AFP level less than or equal to 400 ng/mL, simultaneously. The HC is a more liberal set of criteria for liver transplantation compared with the Milan criteria [17], and most importantly, there were no significant differences in the results for patients fulfilling the Milan or Hangzhou criteria, although the Hangzhou criteria represented a greater tumor burden. There is no research on the discriminating and predictive ability of the HC applied to patients with HCC for hepatectomy.

Generally, patients with resectable HCC have homogenous characteristics and more favorable outcomes than those with unresectable HCC. It is worth determining a potential prognostic staging system to provide these patients with a general idea of survival rates after surgical resection. The aims of this study are to identify independent predictors of survival for HCC patients who have undergone surgical resection, to evaluate the HC and compare them with the BCLC and TNM staging systems, and to identify whether the HC possess the best prognostic value in predicting survival in a large cohort of patients with resectable HCC from a hepatobiliary center in the western part of China.

## Patients and Methods

### Patients

Patients diagnosed with hepatocellular carcinoma and underwent hepatectomy at the Department of Liver Surgery, West China Hospital, Sichuan University from July 2007 to December 2009 were enrolled retrospectively. All patients were confirmed histopathologically by at least two pathologists. A baseline evaluation that included a clinical examination, laboratory studies, and imaging studies (i.e., CT or MRI) was required. The baseline was defined as the time of the last evaluation before partial hepatectomy (PH). The HCC diagnosis was confirmed by histopathological examination of surgical samples. The severity of liver dysfunction was assessed using the Child-Pugh classification. Hepatitis B virus (HBV) infection was tested using the electrochemiluminescence immunoassay (ECLIA) method, and HBsAg positive was defined as HBV infection. The detected serum AFP values ranged from 0 to 1 210.0 ug/L, and all of the AFP values greater than 1 210.0 ug/L were recorded as 1 210.0 ug/L in our hospital. The background information of the patients is listed in Table 1.

**Table 1:** Baseline characteristics and univariate analysis of predictors of survival of the 774 enrolled hepatocellular carcinoma patients.

Variables	No. of patients (%)	MST (month)	*P*-Value
Median age (year, range)	51(18∼87)		0.97
<50	366(47,3)	31	
≥50	408(52.7)	25	
Sex (male/female)			0.564
Male	660(85.3)	29	
Female	114(14.7)	25	
HBsAg(+/−)			
Yes	667(86.2)	24	0.713
No	107(13.8)	30	
Anti-HCV(+/−)			0.656
Yes	44(5.7)	25	
No	730(94.3)	29	
Child–Pugh grade			0.264
A	691(89.2)	30	
B	83(10.8)	21	
Median AFP(ng/mL, range)	307.1(0.6∼1210)		0.014
<8	204(26.4)	38	
8∼400	223(28.8)	29	
>400	347(44.8)	24	
Tumor number			<0.001
1	665(85.9)	35	
2∼3	98(12.7)	18	
≥4	11(1.4)	17	
Tumor size (cm)	6.5(0.5∼22)		<0.001
<5	272(35.1)	54	
5∼8	304(39.3)	29	
>8	198(25.6)	13	
Differentiation of tumor			<0.001
I–II	355(45.9)	51	
III–IV	419(54.1)	12	
Vascular invasion			
Portal vien			<0.001
Yes	264(34.1)	15	
No	510(65.9)	43	
Hepatic vein			<0.001
Yes	58(7.5)	10	
No	716(92.5)	34	
distant Metastasis at diagnosis			<0.001
Yes	5(0.6)	3	
No	769(99.4)	29	
Lymph node metastasis			<0.001
Yes	34(4.4)	11	
No	740(95.6)	31	

AFP, a-fetoprotein; HBV, hepatitis B virus; HCV, hepatitis C virus; MST, mean survival time.

Patients were excluded if data were missing for the classification of patients in any of the three staging systems; patients who received nonsurgical treatment or who received initial treatments for HCC at other hospitals were excluded.

### Tumor stage and follow-up

All patients were retrospectively assigned to the various stages according to the classification criteria of the BCLC, TNM and HC staging systems. Each classification was strictly based on the patients' clinical information. BCLC stage system was derived from pre-operative data of the patients, TNM and HC stage system were derived from post-operative information. Univariate and mutilvariate analysis of predictors of survival of the patients were performed with the data of tumor specimens instead of the radiologic data, like tumor size, tumor number, vascular invasion etc, for sake of inaccurate analysis.

During the first 6 months after surgery, the patients were re-examined every 1–2 months and then every 3–6 months. Clinical, laboratory and radiological (abdominal computed tomography scan and chest X-ray) data were collected at each follow-up. A total of 708 (91.5%) HCC patients were followed until the end of January 2012 or death, while 66 (8.5%) HCC patients were lost during the follow-up period.

### Treatment

All 774 patients underwent a preoperative evaluation before partial liver resection; ultrasonography, computed tomography (CT) scans and magnetic resonance imaging (MRI) were conducted to assess the resectability of tumors and the metastatic focus. Liver function was cautiously evaluated by biochemistry tests and the Child-Pugh classification, as mentioned above. The resection principles were stable and fixed during the research course, including resectable tumor mass, tumor thrombus, metastatic focus and guarantee of adequate liver function reserve. Radiofrequency ablation (RFA) was considered for satellite tumor nodules from the contralateral hepatic lobe in addition to maximum resection of the intrahepatic HCC. Anatomical resection was always the first choice, according to the Couinaud's nomenclature for liver segmentation. Non-anatomical resection with a sufficient resection margin was also adopted for cases in which enough volume of the left liver needed to be preserved. Minor hepatectomy (<3 segments), major hepatectomy (≥3 segments), and wedge resection were performed in 317 (40.9%), 198 (25.6%), and 259 (33.5%) patients, respectively. Prior to surgery for HCC, of the 774 patients, 55 (7.1%) with unresectable HCC underwent surgery after transcatheter hepatic arterial chemoembolization (TACE, down-staged). Moreover, TACE, RFA and biotherapy or traditional Chinese therapy were applied to HCC patients with inoperable intrahepatic recurrence or extrahepatic metastases after surgery.

### Statistical analysis

Survival status after surgery was the end point used to assess the performance of the three staging systems. Length of survival was calculated from the date of surgery to the date of death, or in the case of survivors, to the date of the last follow-up visit. Quantitative data were presented as the mean ± SD. Survival curves for the HCC patients were plotted by the Kaplan-Meier method and compared by the log-rank test for univariate analysis. After the univariate analysis, only variables with P value <0.1 were used in the multivariate analysis, which used the Cox proportional hazard model to identify independent survival predictors. Hazard ratios (HR) and 95% confidence intervals (CI) were calculated. The prognostic performance of a prognostic staging or scoring system was statistically assessed; this performance has been shown to be related to homogeneity (relatively small differences in survival among patients of the same stage by a given system), monotonicity of gradients [12] (survival of patients with earlier stages longer than survival of patients with more advanced stages according to the same system) and discriminatory ability [18] (relatively large differences in survival among patients with different stages by a given system). High homogeneity indicated small differences in survival among patients in the same stages and was determined by the likelihood ratio (LR) χ^2^ test based on a Cox proportional hazard regression model. Monotonicity of gradients represented the overall predictive power of survival for each staging system and the accurate prediction of survival. This point was evaluated by the linear trend χ^2^ test using a Cox regression model. To evaluate the discriminatory ability for the prediction of survival, we evaluated the accuracy of the 1-, 3-, and 5-year mortality predictions for each staging system by calculating the area under the receiver operating characteristic (ROC) curve (AUC) for each staging system. To perform this test, patients censored before 1, 3, and 5 years of follow up were excluded from the analysis. The linear trend χ^2^ method was also used to measure the discriminatory ability of each staging system [19]. The c-indexes (indexes of concordance) were used as parameters of discriminatory ability to investigate the concordance proportion between survival time and stage progression in patients. A multivariate Cox model comprising all three staging systems as covariates was built to evaluate the independent contribution of each staging system to the overall predictive ability for survival by reducing each staging system individually from the whole model and comparing the corresponding values [12], [13]; The AIC is the most important statistical method for comparing different staging systems; when AIC values are lower, the stage is more accurate and informative,higher AIC values following the removal of a model indicated better prognosis capacity of the removed staging system All P values <0.05 were considered statistically significant. Statistical analysis was conducted with the SPSS software package (version 17.0, SPSS, Chicago, IL) or JMP statistical software package, version 4.0 (SAS Institute, Cary, NC, USA).

### Ethical Consensus

Informed consent was obtained from all subjects for participation in the study. The study was approved by the Ethical Committee of West China Hospital and was in accordance with the Helsinki Declaration of 1975. Written informed consent was obtained from the patients or their guardians.

## Results

**Figure 1:**
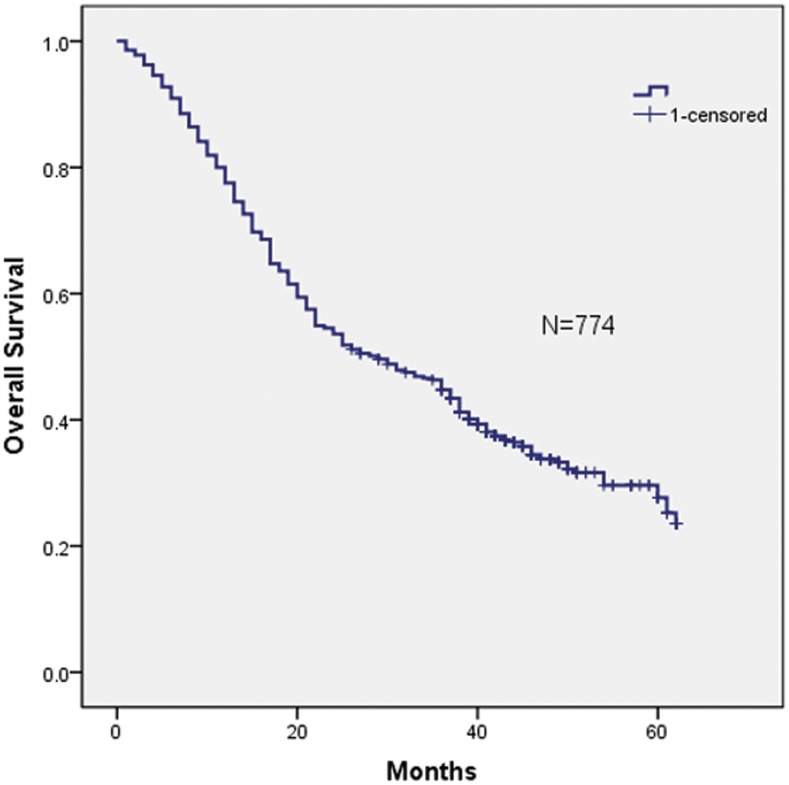
Kaplan-Meier estimated survival curve of 774 HCC-patients after surgical resection. The 1-, 3-, and 5-year overall survival rates were 81.5%, 47.7% and 27.6%, respectively.

Between July 1, 2007 and December 31, 2009, 1857 patients with HCC were seen by medical oncologists at the Department of Liver Surgery, West China Hospital, Sichuan University; of these, 1083 patients were excluded from the study pool because of did not received the hepatectomy or important data missed. The remaining 774 patients were staged using each of the three different staging systems discussed in the methods section. The cohort included 660 male and 114 female patients. The ratio of male to female patients was 5.79 (660/114). The overall mean age was 50.4±12.2 years (range, 18–87 years). The patient baseline characteristics and operative variables are summarized in Table 1. The HBsAg positive rate was 86.2% (n = 667), while the anti-HCV positive rate was 5.7% (n = 44). The tumor was larger than 8 cm in 25.6% (n = 198) of these HCC patients. Macroscopic vascular invasion was present in 322 patients (41.6%), and portal vein invasion (PVT) was present in 264 of these patients. In addition, extrahepatic spread was present in 39 patients (5.03%), including 5 with distant metastasis and 34 with lymph node invasion. The median duration of follow up was 38 months (range, 1–64). At the time of final analysis, 500/774 (64.6%) patients had died of liver disease. The 1-, 3-, and 5-year overall survival rates were 81.5%, 47.7% and 27.6%, respectively (see Figure 1).

### 3.1. Prognostic factors by univariate and multivariate analysis

Univariate analysis identified AFP, maximum tumor size, tumor number, vascular invasion of portal vein and hepatic vein, tumor differentiation, distant organ metastasis and lymph node metastasis as significant predictors of survival in patients with HCC after resection (Table 1). In the multivariate Cox's proportional hazard regression analysis, an all-possible-subset regression approach was used to find the best predictors of survival. Serum AFP level (P = 0.02), tumor size (P<0.001), tumor number (P<0.001), portal vein invasion (P<0.001), hepatic vein invasion (P<0.001), tumor differentiation (P<0.001), distant organ metastasis (P = 0.016) and lymph node metastasis (P<0.001) were independent predictors of the HCC patients' survival among the entire cohort (see Table 2).

**Table 2:** Independent Prognostic Factors for Overall Survival in Patients with HCC According to Multivariate Analysis.

Variables	Rating	HR	95% CI	*P*-value
Tumor number	0 = single; 1 = multiple	1.862	1.229–1.984	<0.001
Tumor size	0 = <8 cm; 1 = ≥8 cm	2.776	2.289–3.367	<0.001
Lymph node metastasis	0 = no; 1 = yes	2.205	1.492–3.261	<0.001
Distant metastasis	0 = no; 1 = yes	3.335	1.247–8.920	0.016
AFP	0 = <400; 1 = ≥400 ng/mL	2.239	1.035–3.483	0.02
Child–Pugh grade	0 = grade A; 1 = grade B	1.120	0.835–1.501	0.448
Differentiation of tumor	0 = I–II; 1 = III–IV	2.492	1.369–6.472	<0.001
Portal vein invasion	0 = no; 1 = yes	1.840	1.624–2.085	<0.001
Hepatic vein invasion	0 = no; 1 = yes	1.903	1.618–2.239	<0.001

AFP, a-fetoprotein; HR, harzard ratio; CI, confidence interval.

### 3.2 Stratification and survival according to the three clinical staging systems

**Figure 2:**
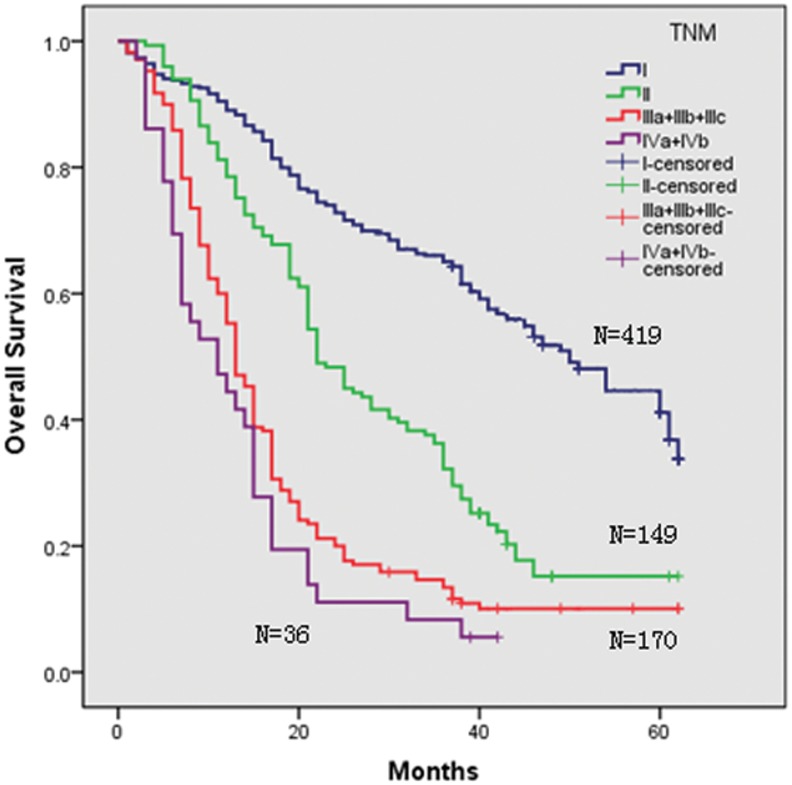
Kaplan-Meier survival analysis stratified according to the TNM staging system.

**Figure 3:**
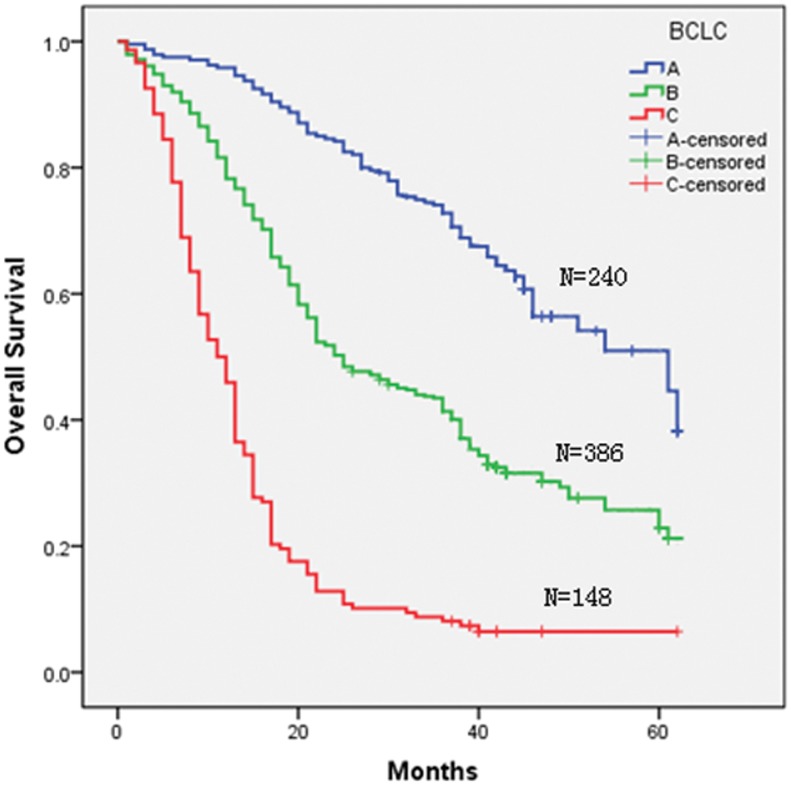
Kaplan-Meier survival analysis stratified according to the BCLC staging system.

**Figure 4:**
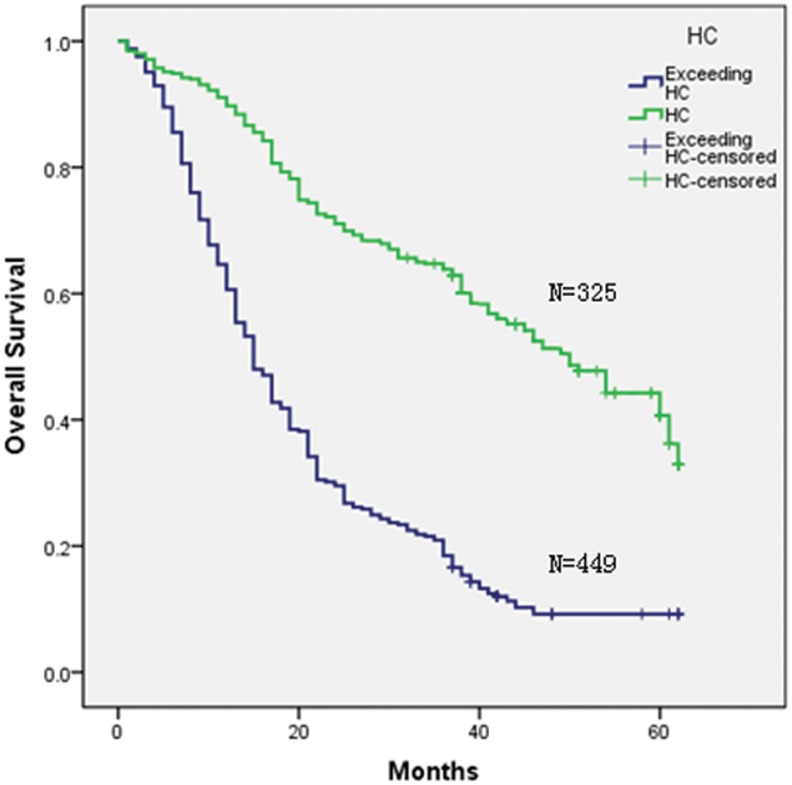
Kaplan-Meier survival analysis stratified according to the Hangzhou Criteria.

Three staging systems were used to stratify the 774 HCC patients into different groups. Kaplan-Meier curves were generated for each of the staging systems; the results are summarized in Figures 1, 2, 3, and 4. The estimated median survival time and the 1-, 3-, and 5-year survival rates based on the different stages are illustrated in Table 3. None of our surgery patients had end-stage disease (BCLC stage D). The Kaplan–Meier survival analysis showed significant differences in the probability of survival for all groups classified with each staging system with high statistical significance (all P<0.001). In addition, significant stratifications of survival were found for all subgroups within two adjacent stages in each staging system (see Table 3).

**Table 3:** Survivals in relation to TNM staging or BCLC and Hangzhou criteria in all patients.

Staging	No. (%)	MST (mo)	1 year(%)	3 years(%)	5 years(%)	HR (95%CI)	*P*	P value between two adjacent stages in a staging system	
TNM							<0.001		
I	419(54.1)	50	89.0	65.1	41.1	1.00(reference)		1 vs. 2	<0.001
II	149(19.3)	22	78.5	32.2	15.2	2.34(1.86–2.94)		2 vs. 3	<0.001
III	170(21.9)	13	55.3	13.4	10.1	4.37(3.52–5.44)		3 vs. 4	0.102
IV	36(4.6)	11	44.4	5.6	0	6.14(4.24–8.89)			
BCLC							<0.001		
A	240(31.0)	61	95.8	72.8	44.6	1.00(reference)		A vs. B	<0.001
B	386(49.9)	25	78.2	41.3	22.9	2.47(1.95–3.12)		B vs. C	<0.001
C	148(19.1)	11	50.0	8.1	0	7.47(5.69–9.79)			
HC							<0.001		
Yes	325(41.9)	50	89.8	63.8	40.7	1.00(reference)			
Exceeding	449(58.1)	15	60.6	18.5	9.2	3.49(2.91–4.19)			

MST, Median survival time; HR, harzard ratio; mo, month.

### 3.3 Prognostic powers of the various staging systems

In agreement with the survival curves described, univariate analyses performed using the linear trend test, the LHR test, and c-indexes demonstrated that the TNM system had a higher prognostic competency than the other two systems in terms of homogeneity (linear χ^2^, 137.523), while BCLC had a higher prognostic competency with respect to discriminatory ability (LHR, 151.119), and the HC had a higher prognostic competency with regard to monotonicity of gradient (c-index, 0.6866), as shown in Table 4.

**Table 4:** Performance of the various TNM staging systems, BCLC and Hangzhou criteria systems.

Staging	Linear trend χ^2^-test	LHR (*P*-value)	C-index
TNM	137.523	150.276	0.6566
BCLC	105.246	151.119	0.6816
HC	116.156	148.934	0.6866
Multivariate model	-Log-likelihood	LHR (P-value)	AIC
Full model	2935.513	279.259(<0.001)	5877.0256
Removing BCLC	2954.072	242.141(<0.001)	5912.1434
Removing TNM	2939.698	270.888(<0.001)	5883.3966
Removing HC	2960.236	229.812(<0.001)	5924.4729

In the univariate model, the highest χ^2^ by the linear trend test, likelihood ratio (LHR) test, and highest c-index were considered to indicate best prognostic performance in terms of discriminatory ability, homogeneity, and monotonicity of gradients. In the multivariate Cox regression model,the independent contributions of each staging system to the full model were evaluated by removing the system concerned. AIC, Akaike information criterion; TNM, tumor-node- etastasis; BCLC, Barcelona Clinic Liver Cancer; HC, Hangzhou criteria.

Moreover, discriminatory ability for mortality at 1, 3, and 5 years, as evaluated by ROC curve area analysis, was the highest for the BCLC staging system (the AUC for 1-year mortality was 0.759) and the TNM staging system (the AUCs for 3- and 5-year mortality were 0.738 and 0.731, respectively) compared with the other staging systems, as shown in Table 5.

**Table 5:** Discriminatory ability for 1-, 3-, and 5-year mortality evaluated by ROC curves for the three staging systems.

Staging	1-year mortality		3-year mortality		5-year mortality	
	AUC	95%CI	AUC	95%CI	AUC	95%CI
TNM	0.719	0.67–0.76	0.738	0.70–0.73	0.731	0.59–0.87
BCLC	0.751	0.71–0.79	0.733	0.69–0.77	0.684	0.48–0.89
HC	0.704	0.66–0.75	0.716	0.68–0.75	0.711	0.56–0.86

ROC, receiver operating characteristic; AUC, area under the curve; TNM, tumor node metastasis; JIS, Japanese Integrated System; BCLC Barcelona Clinic Liver Cancer; HC, Hangzhou Criteria.

For the additive model, the multivariate analysis using Cox's model evaluated the AIC values to compare the relative goodness of fit for the three different staging systems. The model also demonstrated that the HC made the greatest contribution to the prognostic power of the full model that included the three staging systems (LHR χ^2^, 229.812; AIC, 5924.4729; multivariate model in Table 4).

## Discussion

The identification of prognostic factors within a given study is the basis on which all staging systems have been developed. In our study, a wide range of clinical and tumor parameters were identified as prognostic factors by univariate analysis. Moreover, AFP, number of tumor nodes, tumor size, metastasis of distant organs and lymph nodes, and vascular invasion of the portal vein and hepatic vein were proven to be significant predictors of survival in our multivariate analysis. Serum AFP values have repeatedly been identified as a significant prognostic factor in different studies [20]; serum AFP has a strong relationship with HCC differentiation, size, number and vascular invasion. AFP has been used worldwide as the gold standard compared with other serum markers. Some guidelines stress AFP's role in screening, early detection and monitoring prognosis, such as CLIP and the HC. Another clinically related prognosis factor is the Child-Pugh classification. Many centers regard this classification as the indication for surgery, which is limited to Child A patients and some Child B patients. The tumor parameters, including size, number, vascular invasion and metastasis, remained significant in our multivariate analysis and were almost all included in the TNM, BCLC and HC staging systems. Therefore, their significance in our multivariate analysis came as no surprise and is supported by many other studies that show their prognostic importance. The overall median survival was 29 months, and the 5-year overall survival rate was 27.6%. Our survival data are comparable to those of another recent study from northwest China, which showed an overall median survival of 28 months in a group that included more resectable HCC patients. However, the 5-year survival rate of our patients appears slightly low compared with other reported studies with non-Asian populations. A European study reported a 5-year survival rate of 46.9% [21], and another Germany study of HCC patients reported a 5-year survival rate of 38.9% [22]. The difference in survival between Asian and non-Asian populations with HCC may be due to different disease etiology or more severe underlying liver disease. More than 40% of HCC patients in the world are found in China [23]. Chinese HCC patients often have an underlying HBV-infection and tend to be significantly younger than western patients due to transmission of the virus in younger years and the virus' higher ability to promote tumor development in non-cirrhotic livers [24], [25]. Furthermore, reported survival rates for HCC vary significantly depending on the examined study population. The broad range from 8 months (in a largely nonsurgical group) [26] up to 64 months (in a group of resectable patients) [27] can be explained in part by the different degree of selection. Another reason for different survival data may be the bias resulting from comparisons of different time periods. Progress in diagnosis and treatment may have contributed to improved survival in patients with HCC; there are data reporting that the 5-year survival rate of HCC patients has improved over the past 4 decades in the United States from approximately 4% in 1973 to 11.8% in 2001 [28]. This improvement might be attributed to better treatment options and surveillance programs resulting in earlier detection of HCC. Considering these major differences in epidemiology, it becomes clear why the results of a staging system validation study in one geographic region cannot be automatically generalized to another region.

Choosing an appropriate staging system to classify HCC patients into different groups is of great importance for clinicians. A number of investigations have focused on the evaluation of different staging systems [18], [29], [30]. On the one hand, every staging system was initially developed in different, heterogeneous patient cohorts; different studies have demonstrated better performance of the various staging systems in selected groups of HCC patients in selected areas. Most studies from Japan concluded that JIS or modified JIS was the best staging system for their HCC patients. TNM or CLIP was favored in China, Korea and Taiwan as better staging systems. At the same time, most studies from western countries concluded that either BCLC or CLIP was the best staging system for their HCC patients. In other words, it appears that the best staging system for HCC patients is the staging system developed in the HCC patients' own country. On the other hand, because multiple variables affect survival, it is difficult to identify a supreme prognostic system. No consensus on a unified, worldwide HCC staging system has been reached to date. Generally, patients with resectable HCC have homogenous characteristics and more favorable outcomes than those with unresectable HCC. It is worth determining a promising prognostic staging system for those patients eligible for surgery.

In our study, using Kaplan–Meier analysis, we found that all staging systems revealed a progressive decrease in survival from the earliest to the most advanced stage and that all three staging systems showed a significant difference in discriminative capability for survival across different stages (see Table 3); the adjacent stages within the TNM and BCLC staging systems demonstrated obvious discrimination for both early and advanced stages.

A statistical method has been established and used to measure and compare the prognosis power of staging systems, instead of simply comparing the performance of staging system stratification. AIC is regarded as a standard statistical method; the AIC value is considered the most relevant reference for comparing different staging systems. The c-index for the survival analysis model is defined as the probability of concordance given that the pairs considered are usable, meaning that at least one had an event. This index can be interpreted as the probability that a subject from the event group has a higher predicted probability of having an event than a subject from the non-event group. The AIC, as well as the c-index, provide information on the predictive accuracy of a staging system, which exceeds the information that can be derived by simply looking at the number of distinct strata of a staging system. The AIC and c-index have been used in comparative HCC staging system evaluation studies before, but to the best of our knowledge, this is the first validation study to use both tools to evaluate the HC. Our AIC analysis of Cox's analysis showed that HC staging has superior prognostic power compared to the BCLC and TNM staging systems.

The potential reasons for the advantage of the HC over other staging systems include the following. First, the BCLC staging system was established using a large proportion of unresectable HCC patients. BCLC has been externally validated as more effective than other staging systems in western countries [12]. Some large cohort studies have also shown BCLC to be a preferable staging system in Asia [31]. BCLC not only incorporates tumor-related morphology but also liver performance-related parameters; it can be used to guide the choice of treatment, while other staging systems can only be used to predict survival. For this reason, the AASLD [6] endorsed the BCLC staging system. However, in our center, the principal decision for surgical resection for HCC patients is not based solely on the BCLC criteria, and quite a number of studies have shown that surgical resection for HCC patients beyond the BCLC criteria could offer better survival rates [32]–[34]. Thus, if the choice of treatment for HCC is not made according to the BCLC recommendation, the usefulness of predicting the survival by BCLC is compromised. This fact might explain why BCLC was the best staging system for predicting HCC patient survival in mostly western countries but not in eastern countries or in our study.

Although TNM7th staging was developed using a model that included patients who underwent resection, the system only includes tumor size and the invasion of adjacent and distant vessels and of organs other than tumor differentiation. The TNM7th staging system (AJCC) for HCC has been modified several times, and the latest edition was published in 2009. The TNM7th staging classification provides a superior assessment of solid tumors based only on tumor size and the extent of invasion. However, the TNM7th staging system does not consider liver status when classifying patients; cirrhosis, HBV and HCV impact liver function. As a result, the TNM7th's prognostic ability was regarded as limited.

The HC are a more liberal set of criteria for selecting HCC patients for liver transplantation and was introduced in 2008 by Zheng et al. The HC expand the inclusion criteria to a larger scale compared with the Milan criteria, which contributed to a worldwide trend of selecting only early-stage HCC patients for liver transplantation in the last decade. In Zheng's study, there were no significant differences in overall 5-year and tumor-free survival rates for patients fulfilling the Milan or Hangzhou criteria, although the Hangzhou criteria represented a greater tumor burden. Similarly, the 5-year cumulative survival and tumor-free survival were the same for patients with tumors exceeding the Milan criteria or exceeding the HC. Furthermore, a retrospective study from Europe validated the practicality of the HC in French patients. The role of biomarkers was considered in this staging system; an elevated serum AFP level is believed to indicate hepatic regeneration in response to liver injury [35], and previous studies have shown an association between elevated AFP levels and advanced fibrosis in chronic hepatitis. In Lee's [36] study, the serum AFP level was found to be associated with either hepatic necroinflammation or fibrosis, suggesting that this level may indicate the extent of histological changes in the liver. The serum AFP level should be used to improve the accuracy of this system and, more importantly, because it is a simple classification criterion that clinicians can easily understand, remember and apply in daily practice.

There are several potential limitations of our study. First, our study is limited by the retrospective nature of the analysis and the single-institution experience. Second, the majority of our patients had chronic HBV infection other than HCV infection, which is more common in Eastern Asian countries. Our results might need to be evaluated in a large population from European countries and the United States. Third, the distribution of patients in early or advanced stages may have impacted the results. Fourth, the HC emphasized HCC patients who would receive more survival benefits from aggressive surgery, namely, they focused on the patients who were eligible for scheduled surgical resection. We have to admit this may limit its general application.

In conclusion, the TNM score and BCLC stage have limited application in predicting survival for HCC patients undergoing surgical resection. The Hangzhou criteria staging system is a better choice for these patients.
